# Recombinant Follicle-Stimulating Hormone and Luteinizing Hormone Enhance Mitochondrial Function and Metabolism in Aging Female Reproductive Cells

**DOI:** 10.3390/ijms26010083

**Published:** 2024-12-25

**Authors:** Li-Te Lin, Chia-Jung Li, Yi-Shan Lee, Kuan-Hao Tsui

**Affiliations:** 1 Department of Obstetrics and Gynecology, Kaohsiung Veterans General Hospital, Kaohsiung 813, Taiwan; litelin1982@gmail.com (L.-T.L.); sandylee13131@vghks.gov.tw (Y.-S.L.); 2 College of Health and Nursing, Meiho University, Pingtung 912, Taiwan; 3 School of Medicine, College of Medicine, National Sun Yat-Sen University, Kaohsiung 804, Taiwan; 4 School of Medicine, College of Medicine, National Yang-Ming Chiao Tung University, Taipei 112, Taiwan; 5 Institute of Biopharmaceutical Sciences, National Sun Yat-Sen University, Kaohsiung 804, Taiwan; 6 Department of Obstetrics and Gynecology, Taipei Veterans General Hospital, Taipei 112, Taiwan; 7 Department of Medicine, Tri-Service General Hospital, National Defense Medical Center, Taipei 114, Taiwan

**Keywords:** ovarian aging, mitochondrial function, FSH/LH supplementation, reproductive medicine, cellular metabolism

## Abstract

Ovarian aging significantly impacts female fertility, with mitochondrial dysfunction emerging as a key factor. This study investigated the effects of recombinant follicle-stimulating hormone (FSH) and luteinizing hormone (LH) on mitochondrial function and metabolism in aging female reproductive cells. Human granulosa cells (HGL5) were treated with FSH/LH or not. Mitochondrial function was assessed through various assays, including mitochondrial mass, membrane potential, ROS levels, and ATP production. Mitochondrial dynamics and morphology were analyzed using MitoTracker staining. Cellular respiration was measured using a Seahorse Bioenergetics Analyzer. Metabolic reprogramming was evaluated through gene expression analysis and metabolite profiling. In vivo effects were studied using aging mouse oocytes. FSH/LH treatment significantly improved mitochondrial function in aging granulosa cells, increasing mitochondrial mass and membrane potential while reducing ROS levels. Mitochondrial dynamics showed a shift towards fusion and elongation. Cellular respiration, ATP production, and spare respiratory capacity were enhanced. FSH/LH-induced favorable alterations in cellular metabolism, favoring oxidative phosphorylation. In aging mouse oocytes, FSH/LH treatment improved in vitro maturation and mitochondrial health. In conclusion, FSH/LH supplementation ameliorates age-related mitochondrial dysfunction and improves cellular metabolism in aging female reproductive cells.

## 1. Introduction

Ovarian aging represents a natural physiological process characterized by a progressive decline in reproductive function, particularly in women over the age of 35 [1,2]. This decline is marked by a reduction in ovarian reserve, compromised oocyte quality, and an increased incidence of chromosomal abnormalities, collectively contributing to decreased fertility rates and increased risks of infertility and pregnancy complications [2,3,4]. Various factors, including impaired mitochondrial function and metabolism within both oocytes and surrounding granulosa cells, have been implicated in this age-related decline [5,6,7,8]. Mitochondria, crucial for adenosine triphosphate (ATP) production through oxidative phosphorylation, play pivotal roles in cellular energy provision, regulation of metabolism, and management of oxidative stress, thus emerging as central players in reproductive aging. In reproductive cells, mitochondria fulfill critical functions, supporting energy-demanding processes such as oocyte maturation, fertilization, and subsequent embryo development. Dysregulation of mitochondrial activity can precipitate oxidative stress, impaired ATP production, and compromised cellular function, all of which contribute to the process of reproductive aging [9,10,11,12,13].

Follicle-stimulating hormone (FSH) and luteinizing hormone (LH) are key gonadotropins secreted by the anterior pituitary gland, acting synergistically to stimulate follicular growth and maturation within the ovarian microenvironment [14]. FSH exerts its primary influence during the early to mid ovarian antral stage, primarily acting on granulosa cells to promote follicle growth and steroidogenesis [15,16]. LH stimulates androgen production by theca cells and facilitates follicle maturation and ovulation by granulosa cells [17]. The dynamic interplay between FSH and LH is essential for the proper functioning of the ovarian follicles and the menstrual cycle [18,19]. In assisted reproductive technologies (ART), exogenous administration of recombinant FSH and LH has been widely used to stimulate follicular development in women undergoing in vitro fertilization (IVF) procedures. Previous studies have demonstrated the beneficial effects of FSH and LH supplementation on oocyte quality, follicular growth, and pregnancy outcomes, particularly in older women or those with diminished ovarian reserve [20,21,22,23,24,25].

Recognizing the pivotal role of granulosa cells in supporting oocyte growth and maturation, optimizing mitochondrial function within these cells emerges as a promising avenue for enhancing oocyte quality and improving reproductive outcomes in aging women. Hence, this study aims to investigate the impact of recombinant FSH and LH on mitochondrial function and metabolism in aging female reproductive cells, with a specific focus on granulosa cells. We postulate that FSH and LH supplementation will ameliorate mitochondrial function, bolster cellular metabolism, and alleviate age-related mitochondrial dysfunction in granulosa cells, ultimately leading to improvements in oocyte quality and reproductive outcomes.

## 2. Results

### 2.1. FSH/LH Enhances Mitochondrial Function in Aging Granulosa Cells

**Figure 1:**
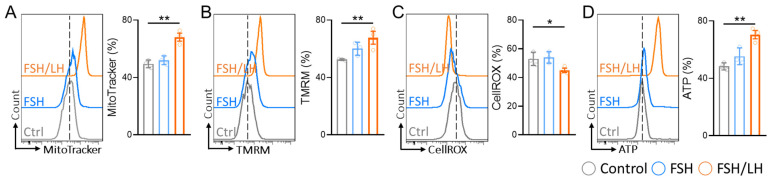
FSH/LH enhanced mitochondrial function in human granulosa cells. We measured (**A**) mitochondrial mass, (**B**) mitochondrial membrane potential, as measured by tetramethylrhodamine methyl ester (TMRM), (**C**) cellular ROS, and (**D**) ATP by flow cytometry. * *p* < 0.05, ** *p* < 0.01.

In our investigation, we aimed to elucidate the superior efficacy of FSH/LH over FSH alone in treating aging cells. Our focus was on scrutinizing alterations in mitochondrial membrane potential (MMP). Notably, cells subjected to FSH/LH treatment exhibited a remarkable retention of approximately 70% of their mitochondrial mass, in stark contrast to the aged group, where cells retained only about 20% (Figure 1A). Moreover, our study demonstrated a significant enhancement in MMP, as measured by tetramethylrhodamine methyl ester (TMRM) fluorescence intensity, in HGL5 cells following FSH/LH treatment (Figure 1B), indicating improved mitochondrial function and cellular energetics. Using CellROS and ATP fluorescent dyes, we investigated the impact of FSH/LH on intracellular hydrogen peroxide levels in aging cells. The results showed a decreasing trend in oxidative stress compared to the control group. However, ATP levels exhibited a significant increase of 20% (Figure 1C,D). In contrast, FSH alone and the control groups exhibited no significant disparities in mitochondrial mass, MMP, reactive oxygen species (ROS), and ATP levels. Consequently, we posit that FSH/LH proves more efficacious in enhancing mitochondrial function compared to the FSH alone group.

### 2.2. FSH/LH Improves the Dynamic Imbalance of Mitochondria in Aging Granulosa Cells

**Figure 2:**
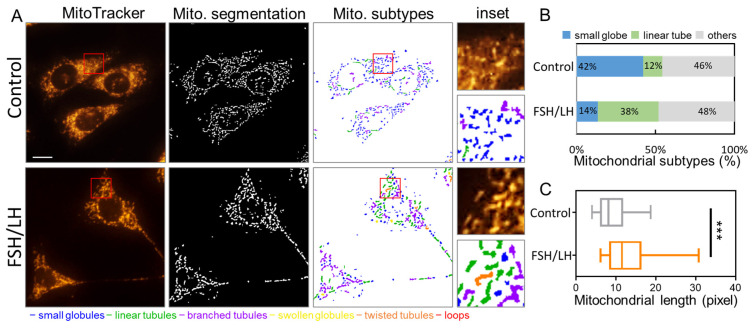
Impact of FSH/LH on mitochondrial dynamic imbalance in aging granulosa cells. (**A**) Assessment and categorization of mitochondrial morphology using MitoTracker fluorescence dye staining. (**B**) Quantification of three primary mitochondrial types: small globule, linear tube, and others. (**C**) Average mitochondrial length in aged granulosa cells before and after treatment. *** *p* < 0.001. Scale bar: 25 μm.

To comprehensively assess the impact of FSH/LH on mitochondrial structure, we employed MitoTracker Red, a mitochondrial staining agent, for visualization purposes. Our findings indicate a significant reduction in mitochondrial debris in senescent cells following treatment with FSH/LH. More than 1000 mitochondria were meticulously examined, and alterations in mitochondrial morphology were categorized using MicroP software [26]. Mitochondria were classified into six distinct groups based on their morphological attributes: globules, linear tubules, loops, branched tubules, swollen globules, and twisted tubules, as depicted in Figure 2A. The data demonstrate a noteworthy 42% decrease in mitochondrial fragmentation, contrasting with a mere 14% reduction in the control group. Moreover, FSH/LH treatment led to a substantial increase in the proportion of elongated mitochondria from 12% to 38%. The remaining four categories were collectively classified as “other” groups (Figure 2B). Furthermore, the average length of mitochondria was markedly greater in the FSH/LH-treated group compared to the control group, as depicted in Figure 2C. These results provide detailed insights into the positive influence of FSH/LH on mitochondrial dynamics, highlighting its potential to counteract age-related mitochondrial imbalance.

### 2.3. FSH/LH Enhances Mitochondrial Biogenesis in Aging Granulosa Cells

**Figure 3:**
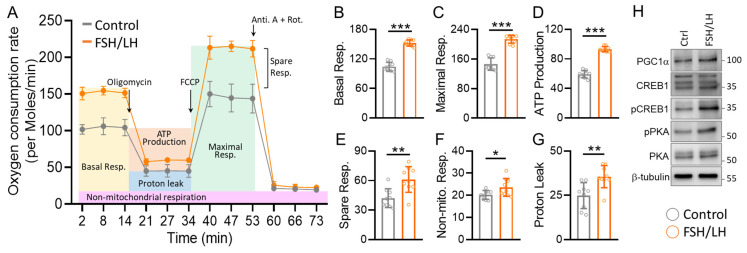
FSH/LH augmented mitochondrial biogenesis in human granulosa cells. (**A**) Evaluation of cellular mitochondrial respiration through oxygen consumption rate (OCR) via the Seahorse Bioscience Analyzer. All OCR values were analyzed for (**B**) basal respiration, (**C**) maximal respiration, (**D**) ATP production, (**E**) spare respiratory capacity, (**F**) non-mitochondrial respiration, and (**G**) proton leakage at different stages. (**H**) Examination of bioenergetic protein levels in granulosa cells via Western blotting. * *p* < 0.05, ** *p* < 0.01, and *** *p* < 0.001.

To gain deeper insights into cellular respiration, we conducted comprehensive mitochondrial function analyses through real-time measurements of oxygen consumption rate (OCR). After stabilizing basal oxygen consumption rates, oligomycin, carbonyl cyanide-4-(trifluoromethoxy)phenylhydrazone (FCCP), and antimycin/rotenone were sequentially introduced. Oligomycin is an ATP synthase inhibitor that blocks the proton channel, thereby inhibiting oxidative phosphorylation and allowing us to measure ATP-linked respiration and proton leak. FCCP is an uncoupling agent that disrupts the MMP by carrying protons across the inner mitochondrial membrane, enabling the assessment of maximal respiratory capacity. Antimycin A and rotenone are inhibitors of complex III and complex I of the electron transport chain, respectively, and their combination allows us to measure non-mitochondrial oxygen consumption. The OCR curves, depicted in Figure 3A, indicated a substantial enhancement in FSH/LH-treated cells compared to the control group. While no difference was observed in basal respiration (Figure 3B), FSH/LH-treated aged cells exhibited statistically significant disparities in maximal respiration (Figure 3C), ATP production (Figure 3D), and spare respiration (Figure 3E). No significant differences were noted in non-mitochondrial respiration or proton leak (Figure 3F,G). Additionally, our study unveiled a significant increase in the expression of bioenergetic proteins related to mitochondrial biogenesis following FSH/LH treatment (Figure 3H). These comprehensive findings suggest a positive impact of FSH/LH on cellular mitochondrial function in aging reproductive cells.

### 2.4. FSH/LH Induces Alterations in the Reprogramming of Cellular Metabolism in Aging Granulosa Cells

**Figure 4:**
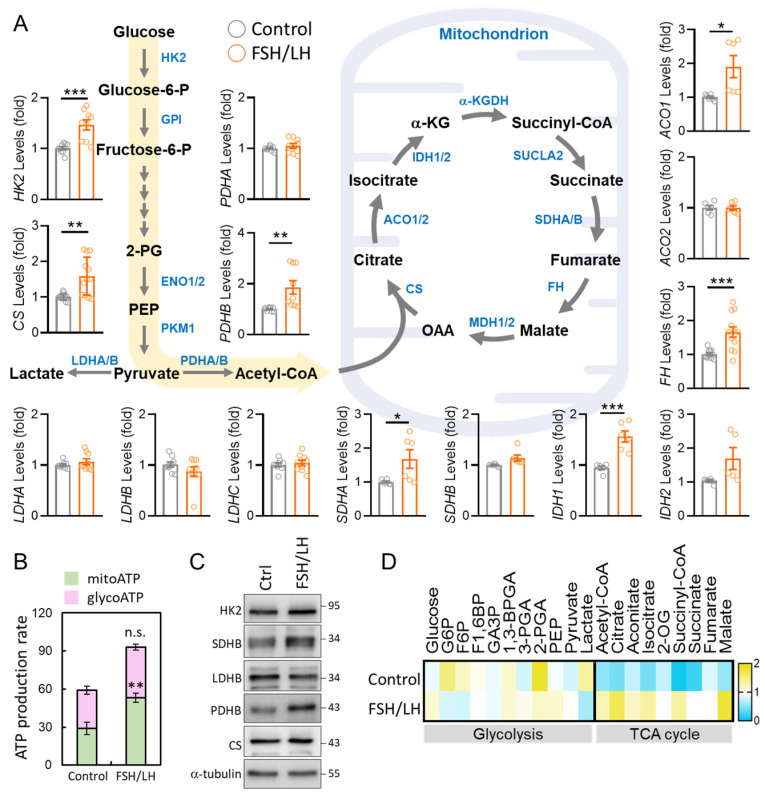
FSH/LH regulated the reprogramming of energy metabolism in aging granulosa cells. (**A**) Evaluation of the expression of FSH/LH-altering metabolic pathways and gene levels involved in glycolysis and the TCA cycle by qPCR analysis. (**B**) Calculation of ATP production rate of aged HGL5 cells supplemented with FSH/LH. (**C**) Assessment of the expression levels of the TCA cycle by Western blotting. (**D**) Metabolite analysis of FSH/LH levels in HGL5 cells.* *p* < 0.05, ** *p* < 0.01, and *** *p* < 0.001.

To assess the impact on aging granulosa cells, we meticulously examined the expression patterns of metabolic genes. Intriguingly, upon FSH/LH administration, there was a remarkable restoration of metabolic gene levels associated with both cellular glycolysis and the tricarboxylic acid (TCA) cycle within the control group. Our comprehensive analysis encompassed genes intricately involved in glycolysis, including hexokinase 2 (HK2), lactate dehydrogenase A/B/C (LDHA/B/C), and pyruvate dehydrogenase A/B (PDHA/B), alongside genes pivotal to the TCA cycle, such as citrate synthase (CS), aconitase 1/2 (ACO1/2), isocitrate dehydrogenase 1/2 (IDH1/2), succinate dehydrogenase complex flavoprotein subunit A/B (SDHA/B), and fumarate hydratase (FH). These findings collectively suggest a profound enhancement in the efficiency of mitochondrial energy transport prompted by FSH/LH. We further employed the ATP rate assay to meticulously track mitochondrial respiration and glycolysis in both control and FSH/LH-supplemented aging granulosa cells (Figure 4A). In the FSH/LH group, a substantial increase in the mitoATP rate, from 28.7% to 53.1%, was observed, contrasting with the absence of a significant difference in glycoATP levels between the two groups. This insightful observation indicates that the augmentation in total ATP in aging cells stimulated by FSH/LH predominantly stems from mitoATP (Figure 4B). Further detailed exploration, employing antibodies, unveiled an FSH/LH-induced upregulation of energy metabolism proteins, which intriguingly coincided with a discernible increase in ATP production (Figure 4C). Simultaneously, we investigated the impact of FSH/LH on glycolysis and the TCA cycle in aging granulosa cell lines. The findings revealed a significant increase in TCA cycle-related metabolite levels following FSH/LH treatment (Figure 4D). This intricate cascade of observations underscores the potential of FSH/LH supplementation to intricately modulate energy metabolism in aging granulosa cells, offering profound insights into the underlying mechanisms governing cellular rejuvenation.

### 2.5. FSH/LH Enhances the Quality of Oocytes in Aging Mice

**Figure 5:**
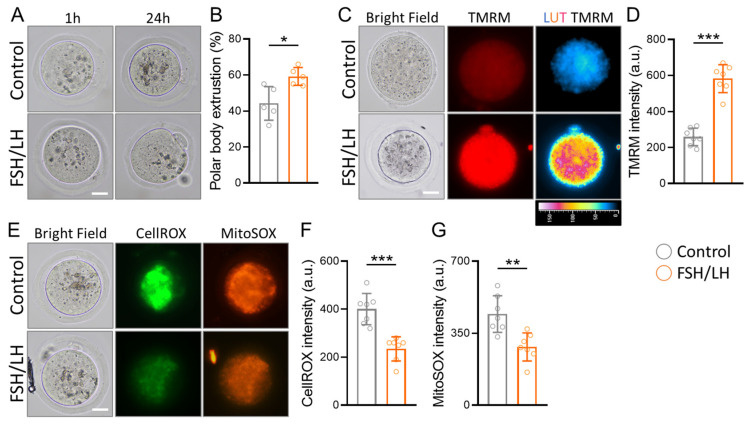
Impact of FSH/LH on in vitro maturation and mitochondrial function in aging mouse oocytes. (**A**) Illustrative images of in vitro maturation of oocytes from aged mice. (**B**) Quantification of the first polar body extruded from mouse oocytes. (**C**) Assessment of mitochondrial membrane potential by TMRM staining to evaluate the effect of the FSH/LH on mouse oocytes. (**D**) Quantification of TMRM red fluorescence intensity. (**E**–**G**) Evaluation of cellular and mitochondrial ROS using CellROX and MitoSOX staining (green and red, respectively) for oocyte quality. Scale bars, 25 µm. * *p* < 0.05, ** *p* < 0.01, *** *p* < 0.001. LUT: Look-up table.

To investigate its impact on the in vitro maturation of mouse oocytes, we assessed maturity by observing the extrusion of the first polar body (PBE). Upon the addition of FSH/LH to the aging oocyte medium and a 24 h incubation, a substantial increase in the number of PBEs and mature oocytes was observed compared to the control group (Figure 5A). Moreover, the results of in vitro maturation after 24 h of FSH/LH exposure demonstrated a significant improvement in PBE (Figure 5B). The oocyte maturation rate in aged mice exhibited a noteworthy enhancement with FSH/LH treatment. To evaluate the efficacy of FSH/LH in ameliorating the poor mitochondrial quality of aging oocytes, we examined MMP as an indicator of mitochondrial health and quality. TMRM-stained oocytes were used to assess the membrane potential, revealing higher red fluorescence intensity in FSH/LH-treated aging oocytes compared to the untreated group (Figure 5C). The decrease in MMP of the FSH/LH-recovered aging oocytes was further demonstrated through pseudo-color visualization of red fluorescence intensity variation and statistical measurements (Figure 5D). Subsequently, we investigated the accumulation of ROS in aging oocytes using two different ROS indicators. The results indicated high ROS accumulation in aging oocytes, but after 24 h of FSH/LH treatment, both markers displayed reduced fluorescence (Figure 5E). The quantification revealed a significant decrease in the intensity of CellROX and MitoSOX fluorescence (Figure 5F,G), underscoring the antioxidative potential of FSH/LH in mitigating oxidative stress in aging oocytes.

## 3. Discussion

The aging process significantly impacts female reproductive function, marked by a decline in oocyte quality and ovarian reserve, leading to reduced fertility rates and increased risks of infertility and pregnancy complications [1,3,4]. Mitochondrial dysfunction has emerged as a critical determinant of age-related reproductive decline, affecting both oocytes and surrounding granulosa cells. Mitochondria play pivotal roles in energy production, metabolism regulation, and oxidative stress management, making them central players in reproductive aging [9,11,12,13]. In this study, we investigated the impact of recombinant FSH and LH on mitochondrial function and metabolism in aging female reproductive cells, particularly granulosa cells. Our findings shed light on the potential of FSH/LH supplementation to ameliorate age-related mitochondrial dysfunction and improve oocyte quality.

One of the key findings of our study is the enhancement of mitochondrial function in aging granulosa cells following FSH/LH treatment, rather than FSH alone. Specifically, FSH/LH supplementation led to a significant increase in mitochondrial mass and membrane potential, indicative of improved mitochondrial health and function. Moreover, FSH/LH treatment resulted in reduced levels of intracellular ROS and increased ATP production, suggesting a reduction in oxidative stress and enhanced cellular energy metabolism. Mitochondrial dysfunction, characterized by decreased MMP and increased oxidative stress, is a hallmark of aging and is closely associated with reproductive decline [13,27]. These results highlight the potential of FSH/LH in ameliorating age-related mitochondrial dysfunction and restoring cellular homeostasis in aging reproductive cells.

Furthermore, our study elucidates the impact of FSH/LH on mitochondrial dynamics in aging granulosa cells. We observed a significant reduction in mitochondrial fragmentation and an increase in mitochondrial length following FSH/LH treatment, indicating a shift towards a more balanced mitochondrial network morphology. Mitochondrial dynamics, including fusion and fission processes, play crucial roles in maintaining mitochondrial homeostasis and function [28,29,30]. Excessive mitochondrial fragmentation is associated with impaired mitochondrial function and cellular dysfunction [31,32,33]. Our findings suggest that FSH/LH promotes mitochondrial fusion and elongation, thereby enhancing mitochondrial dynamics and improving cellular health in aging granulosa cells.

In addition to improving mitochondrial function and dynamics, FSH/LH treatment induced alterations in mitochondrial biogenesis and metabolism in aging granulosa cells. We observed an increase in cellular respiration, ATP production, and spare respiratory capacity in FSH/LH-treated cells compared to untreated aging cells. These findings suggest that FSH/LH enhances mitochondrial biogenesis and metabolic efficiency, leading to increased energy production and cellular function.

Moreover, FSH/LH treatment induced favorable alterations in cellular metabolism in aging granulosa cells, favoring oxidative phosphorylation over glycolysis. This metabolic shift towards increased mitochondrial respiration may contribute to enhanced ATP production and reduced oxidative stress, ultimately improving cellular function and reproductive outcomes in aging women. Dysregulated energy metabolism is implicated in reproductive aging and is associated with mitochondrial dysfunction and oxidative stress [12,27]. The restoration of metabolic gene expression by FSH/LH suggests its potential to normalize energy metabolism and improve cellular function in aging granulosa cells.

Importantly, our study demonstrated the beneficial effects of FSH/LH treatment on oocyte quality in aging mice. FSH/LH treatment improved in vitro oocyte maturation and mitochondrial health, as indicated by increased mitochondrial membrane potential and reduced accumulation of ROS in aging oocytes. Mitochondrial dysfunction and oxidative stress are key determinants of oocyte quality and reproductive aging [5,6,34]. The improvement of mitochondrial health and reduction in oxidative stress by FSH/LH suggest its potential to enhance oocyte quality and reproductive outcomes in aging women.

Prior research has established important roles for FSH and LH in mitochondrial regulation and cellular metabolism in reproductive aging. Lounas et al. demonstrated FSH’s ability to induce rapid mitochondrial restructuring and metabolic reprogramming in porcine cumulus cells [35], while Dong et al. showed FSH alleviates ovarian aging through AMPK-mediated mitophagy to maintain mitochondrial homeostasis and PI3K/AKT-regulated glycophagy to enhance energy metabolism in hens [36]. Additional studies by Jain et al. and Shen et al. revealed FSH’s protective effects against cell death through modulation of apoptotic pathways and oxidative stress responses [37,38]. Regarding LH, Plewes et al. discovered its regulation of mitochondrial dynamics via PKA-DRP1 signaling in luteal cells [39], while Wan et al. demonstrated LH’s impact on granulosa cell gene expression and mitochondrial function [40]. These findings collectively highlight the distinct yet complementary roles of FSH and LH in maintaining mitochondrial health and cellular metabolism in reproductive tissues. However, the synergistic effects of FSH/LH co-treatment on mitochondrial function and bioenergetics in aging reproductive cells remained largely unexplored. Our study provides several novel contributions to the field of reproductive biology. For the first time, we demonstrate the synergistic effects of FSH/LH co-treatment on mitochondrial fusion and biogenesis in aging reproductive cells, revealing their coordinated role in maintaining mitochondrial function. We also uncover the detailed molecular pathways through which FSH/LH enhances spare respiratory capacity, involving activation of specific bioenergetic proteins and metabolic regulators. Moreover, through functional and molecular analyses, we establish the superior efficacy of combined FSH/LH treatment compared to FSH monotherapy in ameliorating age-related mitochondrial dysfunction, highlighting the importance of this dual gonadotropin approach for preserving reproductive cell viability during aging. These findings not only advance our mechanistic understanding but also have important clinical implications for optimizing hormone treatments in reproductive medicine.

The findings of our study have important clinical implications for the management of reproductive aging and infertility. FSH/LH supplementation may offer an approach to improve oocyte quality and reproductive outcomes in aging women undergoing ART. Prior research has shown the advantageous impacts of supplementing with FSH/LH on oocyte quality, follicular development, and pregnancy outcomes, especially in women of advanced age or those with reduced ovarian reserve [20,21,22,23,24]. By enhancing mitochondrial function, dynamics, biogenesis, and metabolism in granulosa cells, FSH/LH treatment may mitigate age-related mitochondrial dysfunction and oxidative stress, thereby improving oocyte quality and pregnancy success rates in aging women.

**Figure 6:**
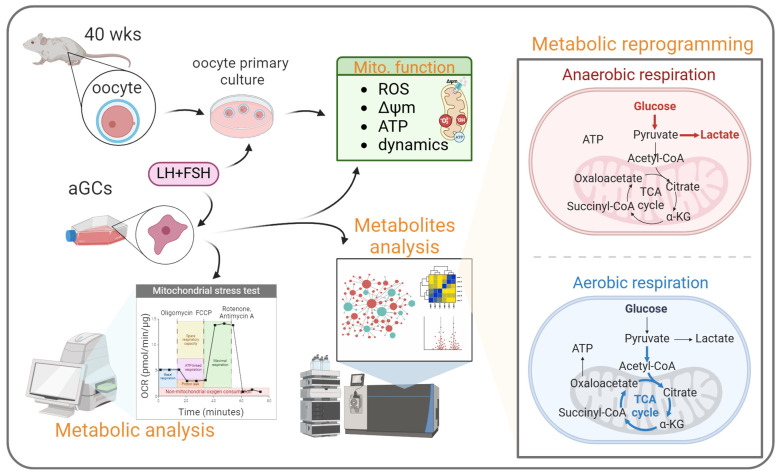
Diagram illustrating the impact of supplementing LH + FSH on the reprogramming of energy metabolism in germ cells.

In summary, our research underscores the advantageous impacts of recombinant FSH and LH on mitochondrial function and metabolism in aging female reproductive cells. FSH/LH holds promise in directly modulating mitochondrial biogenesis, thereby augmenting the capacity of mitochondria to uphold a youthful state in aging cells and decelerating the aging process. By addressing heightened ROS levels in senescent cells, it can proficiently counteract free radicals and bolster oocyte viability. Our investigation utilized FSH/LH to modulate mitochondrial mass and energy metabolism, as depicted in Figure 6. Future studies are needed to explore the physiological mechanisms of FSH/LH, aiming to better understand how to directly or indirectly enhance the antioxidant defense of aging cells or enhance their repair capabilities. The in vitro culture conditions of oocytes can affect factors that hinder cell maturation, making it an important area of clinical artificial reproduction research.

## 4. Materials and Methods

### 4.1. Protocol for Cell Culture and Treatment

The human ovarian granulosa cell line, HGL5, was acquired from Applied Biological Materials Inc. These cells were cultured under controlled conditions in an incubator supplemented with 10% fetal bovine serum (FBS), 1% penicillin/streptomycin, 2% Ultroser G (Pall Corp., Port Washington, NY, USA), and 1% ITS Plus (Zen-Bio Inc., Durham, NC, USA). To establish aging granulosa cells, continuous sub-culturing was performed for over 100 generations, and the aging phenotype was confirmed. The aging cells were then maintained in a humidified incubator at 37 °C with 5% CO_2_. For the experimental procedure, aging granulosa cells were subjected to either the supplemented condition or the absence of supplementation for a duration of 24 h. Subsequent analysis aimed to evaluate the effectiveness of the supplement in promoting germ cell growth. The supplement used in the study consisted of Pergoveris (150 IU), which is a combination of recombinant human follicle-stimulating hormone (hFSH) and human luteinizing hormone (hLH) sourced from Merck Co., Rahway, NJ, USA. Additionally, for cell treatment, FSH was utilized, obtained from GONAL-f^®^ (150 IU), a recombinant human follicle-stimulating hormone (r-hFSH-α) manufactured by Merck Co., Rahway, NJ, USA.

### 4.2. Mitochondrial Function Measurement

Mitochondrial function assays were performed according to established protocols [41]. Cells from each experimental group were harvested and subjected to staining with various fluorescent dyes, including CellROS (5 μM), MitoTracker green (10 nM), tetramethylrhodamine methyl ester (TMRM; 200 nM), MitoSOX (5 μM), and ATP (BioTracker ATP-Red Live Cell Dye, Merck, NJ, USA). Following incubation at 37 °C, excess fluorescent dye was removed by washing the cells with PBS. The resulting cell pellets were then resuspended in PBS for subsequent analysis using flow cytometry. This approach allowed for the comprehensive assessment of mitochondrial function in the different experimental conditions.

### 4.3. Mitochondrial Morphology Analysis

MitoTracker Red CMXRos (Thermo Fisher Scientific Inc., Carlsbad, CA, USA) was diluted to 1 mM using DMSO for mitochondrial morphology analysis. Cells were then incubated with 25–200 nM MitoTracker Red CMXRos for 15 min. After incubation, cells were washed 3 times in prewarmed equilibration medium and imaged immediately or fixed in 4% paraformaldehyde (PFA). After sealing the coverslip, a 40× image capture was performed using the EVOS^®^ FL Cell Imaging System (Thermo Fisher Scientific Inc., Carlsbad, CA, USA).

### 4.4. Oxygen Consumption Rate Measurement

The determination of the oxygen consumption rate followed established procedures [42]. Mitochondrial respiration was assessed using an extracellular flux analyzer and the Seahorse XF HS mini platform (Agilent Technologies, Santa Clara, CA, USA), which measured the oxygen consumption rate (OCR). Cells were seeded in trays and, on average, maintained at a density of 2000 cells per well. Changes in cellular respiration were assessed over time during the mitochondrial function assays, and oligomycin (1 µM), carbonyl cyanide 4-(trifluoromethoxy) phenylhydrazone (FCCP, 0.5 µM), and rotenone (0.5 µM) were administered sequentially. The data obtained were normalized to the cell numbers determined by total protein (O.D.: optical density 595 nm). Each parameter was evaluated in comparison to the corresponding WT cells or without treatment.

### 4.5. Western Blotting Detection

Western blotting was performed as previously described [43]. Total proteins were extracted using RIPA lysis buffer with protease and phosphatase inhibitors, and protein concentrations were measured using a BCA assay to ensure equal loading. Proteins (20–40 µg) were separated by SDS-PAGE and transferred onto PVDF membranes, which were blocked with 5% non-fat milk or BSA for 1 h at room temperature. Membranes were incubated overnight at 4 °C with the following primary antibodies: CREB1 (GTX112846, GeneTex), Phospho-CREB1 (S133) (GTX10564, GeneTex), PGC1α (GTX37356, GeneTex), β-tubulin (GTX628802, GeneTex), PKA RIIα (PRKAR2A; A3889, ABClonal), Phospho-PKA RIIα (S99) (PRKAR2A; AP1034, ABClonal), HK2 (A0994, ABClonal), SDHB (A10821, ABClonal), LDHB (A7625, ABClonal), PDHB (A6943, ABClonal), and CS (A5713, ABClonal). After washing with TBST, membranes were incubated with HRP-conjugated secondary antibodies for 1 h at room temperature. Protein bands were visualized using an ECL detection system, and band intensities were quantified using ImageJ software (NIH, Bethesda, MD, USA). Relative protein expression levels were normalized to β-tubulin, and all experiments were repeated at least three times (*n* = 3–5) to ensure reproducibility.

### 4.6. Animal Experiment and Ovarian Follicle Collection

In this study, C57BL/6J mice sourced from the National Laboratory Animal Center (Taiwan) were employed. These mice were housed under controlled conditions at 25 °C with a 12 h light-dark cycle and provided standard food and water ad libitum. All animal experiments were conducted in accordance with the approved protocols of the Institutional Animal Care and Use Committee (Protocol #2021-2024-A050) of Kaohsiung Veterans General Hospital. For the in vivo component of the study, female C57BL/6J mice aged over 40 weeks were subjected to ovulation induction. This involved an initial injection of 5 IU equine chorionic gonadotropin (eCG), followed by a subsequent injection of 5 IU human chorionic gonadotropin (hCG) after a 48 h interval. Approximately 14–16 h post-hCG injection, the mice were anesthetized, and ovarian follicles were harvested at the cumulus-oocyte complex stage. Subsequently, the cumulus mass was dissociated from the oocytes using a medium containing 0.5 mg/mL hyaluronidase. The isolated oocytes were then cultured at 37 °C in a 5% CO_2_ atmosphere. Following this, the oocytes were randomly allocated into two equal groups: a control group and a treatment group. Oocytes were subjected to an identical concentration of FSH/LH (150 IU) as granulosa cells, aiming to evaluate their physiological function following 24 h of in vitro culture.

### 4.7. Staining of Mouse Oocytes with Fluorophores

To evaluate mitochondrial activity in mouse oocytes, they underwent a 20 min treatment with ICSI Cumulase (Origio, No. 1612, Måløv, Denmark). The resulting cumulus-oocyte complex was incubated in a solution containing CellROX (5 µM) (Molecular Probes) and MitoSOX (5 μM) (Molecular Probes) for 30 min at 37 °C. After thorough washing, the oocytes were examined under a fluorescent microscope to observe their fluorescence intensity.

### 4.8. RNA Extraction and Real-Time Polymerase Chain Reaction (PCR)

Total RNA was extracted from granulosa cells using REzol reagent (Protech Technology), following the manufacturer’s protocol. RNA quantity and quality were assessed using a NanoDrop spectrophotometer, with samples meeting the criteria of A260/A280 ratios between 1.8 and 2.0 selected for further analysis. For gene expression analysis, 1 μg of total RNA was reverse transcribed using HiScript II First Strand cDNA Synthesis Kit (Vazyme). SYBR Green-based quantitative real-time PCR (qRT-PCR) was performed using the StepOne system (Applied Biosystems) (Thermo Fisher Scientific Inc., Carlsbad, CA, USA) with the following cycling conditions: initial denaturation at 95 °C for 10 min, followed by 40 cycles of 95 °C for 15 s and 60 °C for 1 min. Each sample was analyzed in triplicate, and relative gene expression was calculated using the 2−ΔΔCT method, with RNU6-1 serving as the internal reference gene. The specificity of PCR products was confirmed by melting curve analysis. Primer sequences used for real-time PCR are listed in Supplementary Table S1.

### 4.9. Ultra-High Performance Liquid Chromatography–Tandem Mass Spectrometry (UHPLC-MS/MS) Analysis

Metabolite analysis was conducted at the Center for Genomics and Precision Medicine, National Taiwan University, using Ultra-High Performance Liquid Chromatography–Tandem Mass Spectrometry (UHPLC-MS/MS), a highly sensitive and precise analytical technique for metabolite profiling. Sample preparation involved the extraction of metabolites from biological samples, followed by filtration to remove impurities. The prepared samples were then injected into the UHPLC system for separation based on their chemical properties, such as polarity and molecular weight. The separated metabolites were subsequently introduced into the tandem mass spectrometer (MS/MS) for identification and quantification. The MS/MS system employs electrospray ionization (ESI) or atmospheric pressure chemical ionization (APCI) to ionize the metabolites, followed by fragmentation to produce characteristic ion spectra. These spectra were matched against a metabolite database to accurately identify and quantify the target compounds. Data acquisition and analysis were carried out using specialized software for chromatogram processing and peak identification, enabling precise quantification of metabolites. The analysis followed protocols established in our previous study [44], ensuring consistency and reproducibility. Detailed descriptions of the procedures, including sample preparation, chromatographic conditions, and mass spectrometry settings, can be found in the referenced publication.

### 4.10. Statistical Analysis

The results of this study were obtained from at least three independent experiments, with sample sizes ranging from *n* = 5 to 8 for each experimental group. The data are presented as the mean ± standard error of repeated measurements. ImageJ software https://imagej.net/ij/ accessed on 21 December 2024 (NIH, Bethesda, MD, USA), Zenlite 2.1 software (Carl Zeiss Co., Ltd., Jena, Germany), and MicroP software (Taipei, Taiwan) [45] were utilized to characterize and analyze the semi-quantitative and mitochondrial, respectively. The statistical significance was evaluated using GraphPad Prism 8.0 (GraphPad Software, San Diego, CA, USA) and Tukey’s post hoc test was performed to determine the significant differences between group means using a two-way analysis of variance. A *p*-value of less than 0.05 was considered statistically significant.

## Data Availability

The original contributions presented in this study are included in the article/Supplementary Materials. Further inquiries can be directed to the corresponding author.

## Supplementary Materials

The following supporting information can be downloaded at https://www.mdpi.com/article/10.3390/ijms26010083/s1.

## Abbreviations

ATP, adenosine triphosphate; FSH, follicle-stimulating hormone; LH, luteinizing hormone; ART, assisted reproductive technologies; IVF, in vitro fertilization; MMP, mitochondrial membrane potential (MMP); TMRM, tetramethylrhodamine methyl ester; ROS, reactive oxygen species; OCR, oxygen consumption rate; FCCP, carbonyl cyanide-4-(trifluoromethoxy)phenylhydrazone (FCCP); TCA, tricarboxylic acid; HK2, hexokinase 2; LDH A/B/C, lactate dehydrogenase A/B/C; PDHA/B, pyruvate dehydrogenase A/B; CS, citrate synthase; ACO1/2, aconitase 1/2; IDH1/2, isocitrate dehydrogenase 1/2; SDH A/B, succinate dehydrogenase complex flavoprotein subunit A/B; FH, fumarate hydratase; PBE, first polar body.
