# Supplementary material for: Recombinant Follicle-Stimulating Hormone and Luteinizing Hormone Enhance Mitochondrial Function and Metabolism in Aging Female Reproductive Cells
Source: Int J Mol Sci. 2024 Dec 25;26(1):83. doi: 10.3390/ijms26010083 (PMC11720038; doi:10.3390/ijms26010083)
Supplement: Supplementary file 1 [file ijms-26-00083-s001.zip › ijms-3366490-supplementary.pdf]

Table S1. Primer sequences designed for RT-PCR.

**Primers**	Forward	Reverse
HK2	F-TCCGTAACATTCTCATCGATTTCA	R-TGTCTTGAGCCGCTCTGAGAT
LDHA	F-GAAGCGGTTGCAATCTGGAT	R-GGTGAACTCCCAGCCTTTCC
LDHB	F-GGGAACATGGCGACTCAAGT	R-GAGAAACACCTGCCACATTCAC
LDHC	F-GGGCTATTGGACTGTCTGTGATG	R-TGGGTGCACTCTCCTAAGATTTTT
PDHA1	F-ACCCCACAGACCATCTCATCA	R-CCCCGGGTGAAAGTAAAGC
PDHB	F-AACTGTGGTTTCCCATTCAAGAC	R-TTAGATAGCACTGCTGCAGCTTCT
FH	F-CCG TGC CCT CGT TTT GG	R-AAG GAA TTT TGG CTT GCC ATT
CS	F-TCTGGAGCCGAGCCTTAGG	R-GACCCTCTGTGCTCATGGACTT
SDHA	F-TGGGAA AATCAGCAAGCTCTATG	R-ACC ATT CCC CGG TCG AA
SDHB	F-CGCTGCCACACCATCATG	R-CCCTGGATTCAGACCCTTAGC
IDH1	F-CGG AAC CCA AAA GGT GAC AT	R-TGG CAA CAC CAC CAC CTT CT
IDH2	F- CCT GGC GGG CTG CAT	R- GGA AGT GCT CGT TCA GCT TCA
ACO1	F-GGT TTG ACG TGG TGG GCT AT	R-TCA GGT AAA GGC CCA CTG TTG
ACO2	F-TCA ACC CAG AGA CCG ACT ACC T	R-GAG CCT CCA GCC TGA ACT TCT
RNU6-1	F-CTCGCTTCGGCAGCACATATACT	R-ACGCTTCACGAATTTGCGTGT C
